# Comment on ‘Family support for children with learning disabilities to attain good academic performance: A qualitative study’

**DOI:** 10.51866/lte.1124

**Published:** 2026-03-25

**Authors:** MelikŞah Safa ÜÇOK

**Affiliations:** 1 Elbistan State Hospital, Department of Child and Adolescent Psychiatry, Kahramanmaraş, Turkey.

**Keywords:** Specific learning disorder, ADHD, Psychiatric assessment

**Dear Editor,**

I read with great interest the article by Tiengsomboon and Luvira entitled ‘Family support for children with learning disabilities to attain good academic performance: A qualitative study’.^1^ The authors provide a valuable contribution to the literature by focusing on children with learning disabilities (LDs) who demonstrate good academic performance and by exploring family strategies that may support such outcomes. The emphasis on the role of family support offers meaningful insights into potentially protective factors in this population. In this context, a brief consideration of several additional points may further enrich the interpretation of the findings.

One point that may warrant further discussion is the exclusive focus on families of academically successful children with LDs. The absence of a comparison group consisting of children with lower academic performance may limit conclusions regarding the specific contribution of the identified parental approaches to academic success. Including such a comparison in future studies could help clarify whether these family strategies differ meaningfully between groups.

Another aspect that could be considered relates to psychiatric comorbidities commonly associated with specific learning disorders (SLDs). Previous studies have shown that approximately 30%–60% of individuals with SLDs have at least one comorbid psychiatric condition.^2^ Attention-deficit/hyperactivity disorder (ADHD) is reported as the most frequent comorbidity, while anxiety disorders, depressive disorders and other emotional or behavioural difficulties may also co-occur.^3,4^ A review study reported comorbidity rates between SLD and ADHD ranging from 31% to 45%, underscoring the importance of addressing both conditions during clinical assessment and intervention.^5^

Consequently, comorbid psychiatric disorders can affect both intervention planning and academic outcomes. Although students with higher academic performance may present with fewer or less severe comorbidities, comprehensive assessment of psychiatric comorbidity remains an important component of the evaluation and management of all individuals with SLDs. Considering these aspects in assessment and intervention may enhance the delivery of holistic and well-coordinated support for children with SLDs.
